# Dynamic Changes of Metabolite Profiles in Maternal Biofluids During Gestation Period in Huanjiang Mini-Pigs

**DOI:** 10.3389/fvets.2021.636943

**Published:** 2021-07-06

**Authors:** Qian Zhu, Peifeng Xie, Huawei Li, Francois Blachier, Yulong Yin, Xiangfeng Kong

**Affiliations:** ^1^Hunan Provincial Key Laboratory of Animal Nutritional Physiology and Metabolic Process, Key Laboratory of Agro-Ecological Processes in Subtropical Region, National Engineering Laboratory for Pollution Control and Waste Utilization in Livestock and Poultry Production, Institute of Subtropical Agriculture, Chinese Academy of Sciences, Changsha, China; ^2^College of Advanced Agricultural Sciences, University of Chinese Academy of Sciences, Beijing, China; ^3^Université Paris-Saclay, AgroParisTech, INRAE, UMR PNCA, Paris, France; ^4^Research Center of Mini-Pig, Huanjiang Observation and Research Station for Karst Ecosystems, Chinese Academy of Sciences, Guangxi, China

**Keywords:** amino acids, biochemical parameters, biofluids, Huanjiang mini-pigs, gestation

## Abstract

The biochemical parameters related to nitrogenous metabolism in maternal biofluids may be linked and even reflect the fetal metabolism and growth. The present study have measured the concentrations of various parameters related to amino acid (AA) and lipid metabolism, as well as different metabolites including the free AAs in maternal plasma and amniotic and allantoic fluid corresponding to fetuses with different body weight (BW) during different gestation periods, in order to identify the possible relationships between biochemical parameters and fetal growth. A total of 24 primiparous Huanjiang mini-pigs were fed with a standard diet. Data showed that, from day 45 to day 110 of gestation, the maternal plasma levels of alanine aminotransferase (ALT), albumin (ALB), Ile, Orn, Car, α-ABA, and β-AiBA increased (*P* < 0.05); while the levels of ammonia (AMM), choline esterase (CHE), high density lipoprotein-cholesterol (HDL-C), Leu, Glu, Cys, Asp, and Hypro decreased (*P* < 0.05). From day 45 to 110 of gestation, the amniotic fluid levels of aspartate transaminase (AST), CHE, total protein (TP), and urea nitrogen (UN) increased (*P* < 0.05), as well as the level of CHE and TP and concentration of Pro in allantoic fluid; while the amniotic fluid concentrations of Arg, Glu, Orn, Pro, and Tau decreased (*P* < 0.05), as well as allantoic fluid concentrations of Arg and Glu. At day 45 of gestation, the amniotic fluid concentrations of Arg, Orn, and Tau corresponding to the highest BW (HBW) fetuses were higher (*P* < 0.05), whereas the allantoic fluid concentrations of His and Pro were lower (*P* < 0.05) when compared with the lowest BW (LBW) fetuses. At day 110 of gestation, the amniotic fluid concentration of Tau corresponding to the HBW fetuses was higher (*P* < 0.05) than the LBW fetuses. These findings show that the sows display increased protein utilization and decreased lipid metabolism and deposition from day 75 to 110 of gestation. In addition, our data are indicative of a likely stronger ability of HBW fetuses to metabolize protein; and finally of a possible key role of Arg, Gln, Glu, Pro, Tau, and His for the fetal growth and development.

## Introduction

During pregnancy, changing maternal physiological status is associated with significant but reversible metabolic regulation to adapt to the new situation allowing growth and development of fetuses (1, 2). Maternally provisioned environmental conditions and signals affect conceptus, feto-placental, and postnatal development from the time of conception until weaning (3). Suboptimal nutrition affects maternal physiology and fetal growth and development. Optimal fetal growth depends on maternal, placental, and fetal factors, including the external environment; and a genetically predetermined growth potential (4). Inadequate growth and development of fetuses often result in intrauterine growth retardation (IUGR), characterized by fetus body weight (BW) below 10% of the mean BW at the corresponding gestation period (5). IUGR usually lead to alteration of the postnatal growth, together with anomalies related to glucose, cholesterol, and triglyceride metabolism (6, 7). When Huanjiang mini-pigs are fed with diets containing lower nutrient level or with imbalanced diets they display lower BW at first service and bigger litter size, thus explaining why Huanjiang mini-pigs are much susceptible to low birth weight (8).

Fetal growth and development are associated with the fetal nutritional environment (9). Profiling the biochemical parameters (including metabolites like the AAs) in maternal plasma and fetal fluids may reflect the status of growing fetuses, their nutritional requirements and changes in the characteristics of nutrient transport and metabolism (10, 11). The fetal fluids include amniotic fluid and allantoic fluid, both of which have different crucial functions to sustain pregnancy and are vital for fetal growth and development. The amniotic fluid is considered to mirror the intrauterine environment of the fetuses, which provides a unique aqueous environment and is a significant source of nutrients for the fetuses (12). For instance, there are nutrients and growth factors in amniotic fluid, which play important roles in facilitating fetal growth and protecting the fetuses by providing a supportive cushion (13, 14). Although the allantoic fluid is traditionally considered as fetal wastes, it is now clear that it could act as a reservoir for water and other compounds, such as many proteins that can influence fetal-placental development and functions (15). Changes in plasma concentrations of amino acids may play an important role in determining appropriate fetal growth (1). As gestation advanced, the concentrations of biochemical parameters, including free AAs in amniotic and allantoic fluids are known to undergo marked changes (11, 12, 16). Therefore, investigating the changes in these metabolites along gestation is likely to help for a better understanding of the nutritional and metabolic status of fetuses.

The AA composition of the fetal pigs is similar to that of the human fetuses, indicating that the pig is an excellent model for studying AA nutrition and metabolism in the human fetuses (17). In addition, Huanjiang mini-pigs have similar anatomical and physiological characteristics to humans. Lastly, their small size makes their handling easier (18). Therefore, the present study in the Huanjiang mini-pig model, was conducted to investigate the changes of biochemical parameters in maternal plasma, amniotic fluid, and allantoic fluid recovered from fetuses with the highest BW (HBW), middle BW (MBW), or lowest BW (LBW) during different gestation periods, in order to detect the main changes and to propose plausible interpretation in the context of fetal growth and development during gestation.

## Materials and Methods

### Animals, Diets, and Treatments

This study was conducted at the Research Center of Mini-pig, Huanjiang Observation and Research Station for Karst Ecosystems, Chinese Academy of Sciences, Huanjiang, Guangxi, China. A total of 24 primiparous Huanjiang mini-pigs with an initial BW of ~30 kg were reared in eight pens with three pigs per pen. The animals were fed a diet formulated according to the recommendations of the Chinese National Feeding Standard for Swine (Table 1) at 8:00, 15:00, and 18:00 each day, and the feeding quantity was ~2% of maternal BW. The animals were allowed *ad libitum* access to water for the duration of the experiment.

**Table 1:** Ingredients and nutrient levels of the diet (air-dry basis).

**Dietary ingredient**	**Rate (%)**	**Nutrient**	**Level^**b**^**
Corn	54.0	Digestive energy (MJ/Kg)	13.40
Soya meal	12.0	Crude protein (%)	12.04
Rice bran	30.0	Ca (%)	0.78
Premix^a^	4.0	P (%)	0.62
Total	100.0	Lysine (%)	0.53
		Arginine (%)	0.65
		Proline (%)	0.67

a *Provided by per kg premix: VA 301,000 IU, VD 52,800 IU, VE 742 IU, VK_3_ 71 mg, VB_1_ 30 mg, VB_2_ 177 mg, VB_6_ 32 mg, VB_12_ 0.8 mg, nicotinic acid 1,073 mg, D-pantothenic acid 540 mg, folic acid 22.0 mg, biotin 3.0 mg, chorine 8.0 g, Fe 2.0 g, Cu 1.0 g, Zn 3.5 g, Mn 1.3 g, I 14 mg, Co 35 mg, Se 8.3 mg, Ca 200 mg, and P 20 mg*;

b *The values of nutrient levels were calculated*.

### Sample Collection

At days 45, 75, and 110 of gestation, the sows were fasted for 24 h and then weighted. Blood samples were collected by cranial vena cava. Animals were sacrificed under commercial conditions using electrical stunning (120 V, 200 Hz) and exsanguination (19), and then the sows were dissected and the uterus and fetuses were weighed individually. The amniotic fluid and allantoic fluid of each fetus were collected into 10 mL centrifuge tubes. According to the fetal BW per litter, the amniotic fluid and allantoic fluid corresponding to HBW, MBW, or LBW fetuses were selected and stored at −80°C for further analysis.

### Determination of Biochemical Parameters in Plasma, Amniotic Fluid, and Allantoic Fluid

The concentrations of total protein (TP), albumin (ALB), ammonia (AMM), urea nitrogen (UN), total cholesterol (TC), triglyceride (TG), low density lipoprotein-cholesterol (LDL-C), high density lipoprotein-cholesterol (HDL-C), glucose (GLU), total bile acid (TBA), and bilirubin (BIL), as well as the activities of alanine aminotransferase (ALT), aspartate transaminase (AST), alkaline phosphatase (ALP), and choline esterase (CHE) in plasma, amniotic fluid, and allantoic fluid were determined by Cobas c311 Automatic Biochemical Analyzer (Cobas Company, Switzerland). The biochemical kit for BIL was purchased from Beijing Beckman Company, and other biochemical kits were purchased from Cobas Company.

### Determination of Free AA Profiles in Plasma, Amniotic Fluid, and Allantoic Fluid

The free AA profiles in plasma, amniotic fluid, and allantoic fluid of Huanjiang mini-pigs were determined as described previously (20). Briefly, plasma, amniotic fluid, and allantoic fluid were mixed with 8% sulfosalicylic acid in equal proportion, respectively. Then these mixed liquids were stored at 4°C overnight to precipitate protein. The supernatant was filtered through a 0.22-μm membrane into sample bottles after centrifugation at 8,000 × g and 4°C for 10 min. The concentrations of free AAs in plasma, amniotic fluid, and allantoic fluid were determined by an automatic amino acid analyzer (L-8900, Hitachi, Tokyo, Japan).

### Statistical Analysis

The data of sows were analyzed by one-way ANOVA using the SAS version 9.2 (SAS Institute, Inc., Cary, NC, USA). The data of fetuses were analyzed by a mixed-effects model using the SAS version 9.2 and the means were separated using Tukey's method. Results are presented as means plus SEM. Gestation period, BW, and their interactions were included in the statistical model. The effects were considered statistical significance if *P* < 0.05. *P*-values between 0.05 and 0.10 were considered to be trendy.

## Results

### Plasma Biochemical Parameters in Huanjiang Mini-Pigs During Different Gestation Periods

As shown in Table 2, from day 45 to day 110 of gestation, the plasma activity of ALT and concentrations of ALB and TBA increased (*P* < 0.05), while the plasma activity of CHE and concentrations of AMM and HDL-C decreased (*P* < 0.05), and the plasma activity of AST tended to decrease (*P* = 0.0999). At day 75 of gestation, the plasma activity of ALP was the highest (*P* < 0.05) and plasma concentrations of TG and BIL were the lowest (*P* < 0.05), when compared with day 45 and day 75 of gestation.

**Table 2:** Biochemical parameters in plasma of Huanjiang mini-pigs at different gestation periods.

**Items**	**Day of gestation**			**SEM**	***P*-values**
	**45**	**75**	**110**		
ALB, g/L	40.76^c^	46.78^b^	54.69^a^	0.64	<0.0001
ALP, U/L	122.50^a^	133.33^a^	86.20^b^	1.48	<0.0001
ALT, U/L	41.50^c^	54.63^b^	62.27^a^	0.82	<0.0001
AMM, μmol/L	301.90	203.57	185.20	2.96	0.01
AST, U/L	64.17	50.87	47.88	1.37	0.10
BIL, μmol/L	0.72^a^	0.47^b^	0.58^a^^b^	0.16	0.01
CHE, U/L	594.83^a^	496.67^b^	449.83^c^	2.07	<0.0001
GLU, mmol/L	6.45	5.83	5.03	0.52	0.45
HDL-C, mmol/L	1.05	0.94	0.89	0.12	0.05
LDL-C, mmol/L	1.05	1.04	0.92	0.18	0.58
TBA, μmol/L	6.77^b^	7.28^b^	18.78^a^	0.63	<0.0001
TC, mmol/L	2.14	2.03	1.94	0.18	0.37
TG, mmol/L	0.61^b^	0.45^c^	0.80^a^	0.10	<0.0001
TP, g/L	75.71	75.56	73.41	0.72	0.50
UN, mmol/L	3.41	3.46	3.44	0.32	0.99

a−c *Values within a row without a common superscript letter differ (P < 0.05). At days 45, 75, and 110 of gestation, n = 8, 8, and 7 per group, respectively. ALB, Albumin; ALP, alkaline phosphatase; ALT, alanine aminotransferase; AMM, ammonia; AST, aspartate transaminase; BIL, bilirubin; CHE, choline esterase; GLU, glucose; HDL-C, high density lipoprotein-cholesterol; low LDL-C, density lipoprotein-cholesterol; TBA, total bile acid; TC, total cholesterol; TG, triglyceride; TP, total protein; and UN, urea nitrogen. The same as below*.

### Amniotic Fluid Biochemical Parameters in Huanjiang Mini-Pigs During Different Gestation Periods

As shown in Table 3, amniotic fluid AST activity and UN concentration regardless of the BW increased (*P* < 0.05), whereas amniotic fluid HDL-C concentration corresponding to MBW and LBW fetuses decreased (*P* < 0.05) from day 45 to day 110 of gestation. When compared with day 45 of gestation, amniotic fluid ALB concentration, regardless of the BW, decreased (*P* < 0.05) at day 75 and day 110 of gestation, as well as TBA corresponding to MBW and HBW fetuses, LDL-C corresponding to LBW and HBW fetuses, and HDL-C and TC corresponding to HBW fetuses. When compared with day 45 and day 75 of gestation, amniotic fluid CHE activity regardless of the BW increased (*P* < 0.05) at day 110 of gestation, as well as AMM concentration corresponding to LBW and MBW fetuses, and TBA and BIL corresponding to LBW fetuses. The amniotic fluid TP concentration corresponding to three different BW fetuses was the lowest (*P* < 0.05) at day 75 of gestation, as well as TC corresponding to LBW fetuses when compared with day 45 and day 110 of gestation.

**Table 3:** Biochemical parameters in amniotic fluid of fetal pigs with LBW, MBW, and HBW at different gestation periods.

**Items**	**Day 45 of gestation**			**Day 75 of gestation**			**Day 110 of gestation**			**SEM**	***P*****-values**		
	**LBW^**1**^**	**MBW^**2**^**	**HBW^**3**^**	**LBW**	**MBW**	**HBW**	**LBW**	**MBW**	**HBW**		**Gestation period**	**Body weight**	GP*BW
ALB, g/L	3.40^a^	3.29^a^	3.21^a^	2.46^b^	2.68^b^	2.45^b^	2.74^b^	2.45^b^	2.53^b^	0.21	<0.0001	0.4932	0.5169
AMM, μmol/L	156.70^d^	165.08^d^	213.37^cd^	117.75^d^	109.34^d^	125.82^d^	948.56^b^	2309.48^a^	338.67^c^	3.77	<0.0001	<0.0001	<0.0001
AST, U/L	6.73^f^	8.78^f^	7.33^f^	16.43^e^	23.47^d^	17.46^e^	40.88^a^	35.67^b^	30.75^c^	0.65	<0.0001	0.0018	0.0001
BIL, μmol/L	0.95^cd^	1.32^c^	1.03^cd^	0.70^d^	0.68^d^	0.53^d^	3.60^b^	4.33^a^	0.60^d^	0.25	<0.0001	<0.0001	<0.0001
CHE, U/L	50.50^cd^	44.38^cd^	53.67^cd^	56.25^cd^	40.80^d^	43.75^cd^	102.25^b^	154.75^a^	64.00^c^	1.41	<0.0001	0.0211	<0.0001
GLU, mmol/L	1.58	1.69	1.62	2.24	1.68	1.66	1.81	2.20	1.57	0.26	0.2987	0.3432	0.2136
HDL-C, mmol/L	0.05^b^	0.07^a^	0.08^a^	0.03^cd^	0.04^bc^	0.02^de^	0.003^e^	0.003^e^	0.018^e^	0.04	<0.0001	0.0744	0.0008
LDL-C, mmol/L	0.04^a^	0.04^a^	0.04^a^	0.02^bc^	0.02^b^	0.01^bc^	0.005^c^	0.005^c^	0.01^bc^	0.04	<0.0001	0.468	0.6474
TBA, μmol/L	3.17^d^	7.78^a^	7.98^a^	3.97^cd^	5.32^bc^	5.92^b^	6.05^b^	4.25^bcd^	4.38^bcd^	0.44	0.0073	0.0002	<0.0001
TC, mmol/L	0.18^bc^	0.18^bc^	0.19^a^^b^	0.14^d^	0.17^bcd^	0.14^cd^	0.19^a^^b^	0.21^a^	0.16^bcd^	0.06	<0.0001	0.2219	0.0600
TP, g/L	2.28^cd^	2.28^cd^	2.30^cd^	1.37^e^	1.67^de^	1.22^e^	4.40^a^	3.30^b^	2.60^bc^	0.30	<0.0001	0.1238	0.0221
UN, mmol/L	2.44^d^	2.53^d^	2.67^d^	4.80^cd^	4.60^cd^	5.81^c^	15.08^a^	10.63^b^	13.68^a^	0.49	<0.0001	0.1239	0.0536

1 *LBW, the lowest body weight*;

2 *MBW, the middle body weight*;

3 *HBW, the highest body weight*;

a−f *Values within a row without a common superscript letter differ (P < 0.05). Probability values between 0.05 and 0.10 were considered to be trends. At day 45 and day 75 of gestation, in the LBW, MBW, and HBW groups, n = 8 per group; at day 110 of gestation, in the LBW, MBW, and HBW groups, n = 5. The same as below*.

At day 45 of gestation, MBW and HBW fetuses presented higher (*P* < 0.05) amniotic fluid concentrations of HDL-C and TBA compared with the LBW fetuses. At day 75 of gestation, MBW fetuses presented higher (*P* < 0.05) amniotic fluid activity of AST compared with the LBW and HBW fetuses. In addition, MBW fetuses presented higher (*P* < 0.05) amniotic fluid concentration of HDL-C compared with the HBW fetuses; and HBW fetuses presented higher (*P* < 0.05) amniotic fluid concentration of TBA compared with the LBW fetuses. At day 110 of gestation, LBW fetuses presented the highest (*P* < 0.05) amniotic fluid activity of AST, as well as CHE in MBW fetuses; and MBW fetuses presented the highest (*P* < 0.05) amniotic fluid concentration of AMM, as well as BIL in HBW fetuses.

There were interaction effects (*P* < 0.05) between gestation period and BW on the amniotic fluid activities of AST and CHE and amniotic fluid concentrations of TP, AMM, HDL-C, TBA, and BIL.

### Allantoic Fluid Biochemical Parameters in Huanjiang Mini-Pigs During Different Gestation Periods

As shown in Table 4, from day 45 to day 110 of gestation, the allantoic fluid concentration of BIL corresponding to LBW and MBW fetuses increased (*P* < 0.05), as well as GLU corresponding to HBW fetuses; allantoic fluid concentration of AMM corresponding to three different BW fetuses decreased (*P* < 0.05), as well as allantoic fluid activity of AST corresponding to LBW fetuses. When compared with day 45 and day 75 of gestation, allantoic fluid CHE activity and TP and TBA concentrations corresponding to three different BW fetuses increased (*P* < 0.05) at day 110 of gestation, as well as BIL corresponding to HBW fetuses. The allantoic fluid concentration of UN corresponding to three different BW fetuses decreased (*P* < 0.05) at day 45 of gestation; whereas AST activity and HDL-C concentration corresponding to HBW fetuses increased (*P* < 0.05) when compared with day 75 and day 110 of gestation. The allantoic fluid activity of ALT corresponding to LBW fetuses decreased (*P* < 0.05) at day 45 of gestation compared with day 75 of gestation.

**Table 4:** Biochemical parameters in allantoic fluid of fetal pigs with LBW, MBW, and HBW at different gestation periods.

**Items**	**Day 45 of gestation**			**Day 75 of gestation**			**Day 110 of gestation**			**SEM**	***P*****-values**		
	**LBW^**1**^**	**MBW^**2**^**	**HBW^**3**^**	**LBW**	**MBW**	**HBW**	**LBW**	**MBW**	**HBW**		**Gestation period**	**Body weight**	GP*BW
ALB, g/L	3.57^a^	3.02^a^^b^	3.43^a^^b^	3.24^a^^b^	2.89^a^^b^	2.58^b^	3.04^a^^b^	2.90^a^^b^	2.63^b^	0.29	0.0569	0.1275	0.6234
ALT, U/L	3.77^cd^	3.90^cd^	2.98^cde^	6.60^b^	1.28^e^	2.62^de^	4.93^bc^	9.84^a^	2.78^de^	0.45	<0.0001	<0.0001	<0.0001
AMM, mmol/L	1.44^bc^	2.20^a^	2.17^a^	0.78^de^	1.56^b^	1.24^bc^	0.99^bcd^	1.00^cd^	0.53^e^	0.16	<0.0001	0.0006	0.0005
AST, U/L	130.74^a^	89.14^bc^	123.54^a^^b^	83.95^c^	14.40^d^	38.76^d^	45.37^d^	87.63^bc^	25.78^d^	2.00	<0.0001	0.0167	0.0003
BIL, μmol/L	0.15^e^	0.28^de^	0.48^cde^	1.00^cd^	1.19^c^	0.65^cde^	5.15^a^	5.20^a^	4.20^b^	0.28	<0.0001	0.0882	0.1342
CHE, U/L	70.00^c^	58.33^c^	47.25^c^	69.00^c^	60.40^c^	46.20^c^	274.50^a^	228.00^b^	226.00^b^	1.96	<0.0001	0.0151	0.5965
GLU, mmol/L	0.78^d^	0.70^d^	0.60^d^	1.79^bc^	0.87^d^	1.56^c^	2.39^a^	2.26^a^^b^	1.91^abc^	0.24	<0.0001	0.0245	0.0356
HDL-C, μmol/L	28.33^bc^	0.00^d^	58.33^a^	34.29^b^	23.75^bc^	28.75^bc^	13.33^bcd^	10.00^cd^	20.00^bc^	6.37	0.0218	0.0003	0.0011
LDL-C, μmol/L	25.00^b^	5.00^b^	28.33^a^^b^	17.14^b^	13.75^b^	52.50^a^	11.67^b^	16.67^b^	50.00^a^	7.95	0.3409	<0.0001	0.2677
TBA, μmol/L	1.20^c^	1.23^c^	1.32^c^	1.98^c^	1.63^c^	1.40^c^	9.18^a^	8.90^a^	7.35^b^	0.31	<0.0001	0.0108	0.0238
TC, mmol/L	0.17^a^^b^	0.13^a^^b^	0.16^a^^b^	0.21^a^^b^	0.11^b^	0.15^a^^b^	0.26^a^	0.24^a^^b^	0.22^a^^b^	0.11	0.0237	0.2667	0.8834
TP, g/L	4.48^b^	3.85^b^	4.22^b^	3.28^b^	4.14^b^	3.44^b^	6.98^a^	8.55^a^	7.00^a^	0.49	<0.0001	0.5071	0.6598
UN, mmol/L	5.88^c^	6.20^c^	6.00^c^	10.80^b^	15.17^a^	18.02^a^	10.70^b^	18.42^a^	18.20^a^	0.64	<0.0001	<0.0001	0.0189

1 *LBW, the lowest body weight*;

2 *MBW, the middle body weight*;

3 *HBW, the highest body weight*;

a−e *Values within a row without a common superscript letter differ (P < 0.05). Probability values between 0.05 and 0.10 were considered to be trends. At day 45 and day 110 of gestation, in the LBW, MBW, and HBW groups, n = 6 per group; at day 75 of gestation, in the LBW, MBW, and HBW groups, n = 8. The same as below*.

At day 45 of gestation, MBW fetuses presented the lowest (*P* < 0.05) allantoic fluid concentration of HDL-C; MBW fetuses presented lower (*P* < 0.05) allantoic fluid activity of AST; and MBW and HBW fetuses presented higher (*P* < 0.05) allantoic fluid concentration of AMM, when compared with the LBW fetuses. At day 75 of gestation, HBW fetuses presented higher (*P* < 0.05) allantoic fluid concentration of LDL-C compared with the LBW and MBW fetuses. MBW and HBW fetuses presented lower (*P* < 0.05) allantoic fluid activity of ALT, while presenting higher (*P* < 0.05) allantoic fluid AST activity and AMM and UN concentrations, when compared with the LBW fetuses. In addition, HBW fetuses presented higher (*P* < 0.05) allantoic fluid concentration of GLU compared with the MBW fetuses. At day 110 of gestation, MBW fetuses presented the highest (*P* < 0.05) allantoic fluid activity of ALT. HBW fetuses presented higher (*P* < 0.05) allantoic fluid concentration of LDL-C, whereas this group of animals presented lower (*P* < 0.05) allantoic fluid concentrations of AMM, TBA, and BIL, when compared with the LBW and MBW fetuses. Lastly, MBW and HBW fetuses presented lower (*P* < 0.05) allantoic fluid activity of CHE, while presenting higher (*P* < 0.05) allantoic fluid concentration of UN, when compared with the LBW fetuses; and finally LBW and HBW fetuses presented lower (*P* < 0.05) allantoic fluid activity of AST compared with the MBW fetuses.

There were interaction effects (*P* < 0.05) between gestation period and BW on the allantoic fluid activities of ALT and AST, and allantoic fluid concentrations of AMM, UN, HDL-C, GLU, and TBA.

### Plasma Free AA Profiles in Huanjiang Mini-Pigs During Different Gestation Periods

As shown in Table 5, from day 45 to day 110 of gestation, the plasma concentrations of Ile, Car, Cysthi, Orn, 3-Mehis, α-AAA, and β-AiBA increased (*P* < 0.05), whereas those of Asp, Cys, Glu, Hypro, Pro, PEA, and Sar decreased (*P* < 0.05). The plasma concentrations of Lys, Val, EOHNH2, Hylys, Tau, 1Mehis, and β-Ala were the highest (*P* < 0.05), whereas those of Leu, Phe, Cit, Tyr, and α-ABA were the lowest (*P* < 0.05) at day 75 of gestation.

**Table 5:** Free AA profiles in plasma of Huanjiang mini-pigs at different gestation periods (nmol/ml).

**Items**	**Day of gestation**			**SEM**	***P*-values**
	**45**	**75**	**110**		
1-Mehis	7.58^c^	11.04^a^	8.67^b^	0.25	<0.0001
3-Mehis	10.06^b^	11.23^b^	15.96^a^	0.43	<0.0001
Ala	606.00	570.27.	570.90	2.30	0.1871
Arg	186.04	178.04	168.23	1.76	0.3980
Asp+Asn	23.24^a^	20.58^b^	15.20^c^	0.51	<0.0001
Car	32.35^b^	36.25^a^^b^	41.21^a^	0.86	0.0302
Cit	114.71^a^	93.80^b^	101.59^a^^b^	1.53	0.1015
Cys	71.93^a^	53.52^b^	53.63^b^	0.90	<0.0001
Cysthi	3.51^c^	11.35^b^	13.38^a^	0.24	<0.0001
EOHNH2	10.27^b^	18.00^a^	17.58^a^	0.35	<0.0001
Glu+Gln	289.42^a^	286.77^a^	217.55^b^	1.68	<0.0001
Gly	843.50^b^	956.10^a^	922.33^a^^b^	3.33	0.0546
His	47.33^a^^b^	46.25^b^	49.50^a^	0.57	0.0750
Hylys	5.32^c^	65.87^a^	59.97^b^	0.59	<0.0001
Hypro	59.69^a^	19.46^b^	20.45^b^	0.50	<0.0001
Ile	94.01^c^	107.24^b^	112.23^a^	0.71	<0.0001
Leu	226.22^a^	203.22^b^	212.20^b^	1.26	0.0062
Lys	104.22^b^	178.94^a^	168.17^a^	1.52	<0.0001
Met	38.62	42.29	43.18	0.88	0.3256
Orn	88.91^b^	101.94^a^	101.50^a^	1.10	0.0242
PEA	14.78^a^	13.94^a^	10.84^b^	0.58	0.0246
Phe	85.89^a^	81.21^b^	84.71^a^	0.59	<0.0001
Pro	304.84^a^	41.28^b^	38.76^b^	1.53	<0.0001
P-Ser	8.51^a^	8.32^a^^b^	7.93^b^	0.25	0.0879
Sar	36.24^a^	29.59^b^	29.61^b^	0.63	0.0004
Ser	143.16^a^	126.63^b^	140.27^a^^b^	1.33	0.0669
Tau	110.10^b^	132.87^a^	123.64^a^	1.26	0.0064
Thr	186.03	181.26	182.96	1.39	0.8245
Tyr	100.42^b^	71.77^c^	126.34^a^	1.01	<0.0001
Val	240.99^c^	285.20^a^	262.28^b^	1.43	<0.0001
α-AAA	24.10^b^	30.02^b^	36.72^a^	0.88	0.0035
α-ABA	12.32^b^	11.60^b^	19.81^a^	0.52	<0.0001
β-AiBA	0.43^c^	0.79^b^	0.94^a^	0.13	<0.0001
β-Ala	7.22^b^	8.03^a^	7.18^b^	0.29	0.0418
γ-ABA	0.39	0.40	0.37	0.14	0.9318

a−c *Values within a row without a common superscript letter differ (P < 0.05). Probability values between 0.05 and 0.10 were considered to be trends. 1-Mehis, 1-methyl-histidine; 3-Mehis, 3-methyl-histidine; Ala, alanine; Arg, arginine; Asp, aspartate; Asn, asparagine; Ans, anserine; Car, carnosine; Cit, citrulline; Cys, cysteine; Cysthi, cystathionine; EOHNH2, ethanolamine; Glu, glutamate; Gln, glutamine; Gly, glycine; His, histidine; Hylys, hydroxy-lysine; Hypro, hydroxy-proline; Ile, isoleucine; Leu, leucine; Lys, lysine; Met, methionine; Orn, ornithine; Phe, phenylalanine; Pro, proline; P-Ser, O-phosphos-serine; Sar, sarcosine; Ser, serine; Tau, taurine; Thr, threonine; Tyr, tyrosine; Val, valine; α-AAA, α-aminoadipic acid; α-ABA, α-amino-n-butyric acid; β-AiBA, β-aminoisobutyric acid; β-Ala, β-alanine; and γ-ABA, γ-amino-n-butyric acid. The same as below*.

### Amniotic Fluid Free AA Profiles in Huanjiang Mini-Pigs During Different Gestation Periods

As shown in Table 6, from day 45 to day 110 of gestation, amniotic fluid concentration of His corresponding to MBW and HBW fetuses increased (*P* < 0.05), as well as those of Hypro and α-AAA corresponding to MBW fetuses. Amniotic fluid concentrations of Glu and Pro corresponding to three different BW fetuses decreased (*P* < 0.05), as well as that of Orn corresponding to LBW and MBW fetuses and those of Arg and Tau corresponding to MBW fetuses. Amniotic fluid concentration of Cit corresponding to the three different groups of fetuses with different BW were the highest (*P* < 0.05), as well as that of His in the MBW and LBW groups of fetuses at day 75 of gestation. Amniotic fluid concentration of Phe corresponding to three different groups of fetuses with different BW was the lowest (*P* < 0.05), as well as Val in the LBW and HBW groups of fetuses, and Met in the LBW group of fetuses at day 75 of gestation. When compared with day 45 and day 75 of gestation, amniotic fluid concentrations of EOHNH2, PEA, β-AiBA, and γ-ABA corresponding to three different BW fetuses increased (*P* < 0.05) at day 110 of gestation, as well as Thr and α-AAA corresponding to LBW and HBW groups of fetuses, Gly, P-Ser, and β-Ala corresponding to LBW and MBW fetuses, and Leu, Asp, Cysthi, Ser, Tyr corresponding to LBW fetuses. When compared with day 75 and day 110 of gestation, amniotic fluid concentrations of Ile, Lys, and Ala corresponding to the three different groups of fetuses with different BW increased (*P* < 0.05) at day 45 of gestation, as well as Arg and Tau in the groups corresponding to LBW and HBW fetuses, and Tyr and Orn corresponding to HBW fetuses; whereas amniotic fluid concentrations of Car and Hypro corresponding to LBW and HBW fetuses decreased (*P* < 0.05) at day 45 of gestation. When compared with day 75 of gestation, amniotic fluid concentration of Ser corresponding to HBW fetuses increased (*P* < 0.05) at day 45 of gestation.

**Table 6:** Free AA profiles in amniotic fluid of fetal pigs with LBW, MBW, and HBW at different gestation period (nmol/ml).

**Items**	**Day 45 of gestation**			**Day 75 of gestation**			**Day 110 of gestation**			**SEM**	***P*****-values**		
	**LBW^**1**^**	**MBW^**2**^**	**HBW^**3**^**	**LBW**	**MBW**	**HBW**	**LBW**	**MBW**	**HBW**		**Gestation period**	**Body weight**	GP*BW
Ala	744.06^a^	631.61^b^	798.36^a^	484.65^cd^	483.69^cd^	531.08^bc^	384.08^d^	432.26^cd^	610.49^b^	3.45	<0.0001	0.0001	0.0312
Ans	6.83^c^	7.46^c^	7.19^c^	8.45^c^	12.49^c^	13.74^c^	434.42^a^	171.59^b^	63.00^c^	2.88	<0.0001	<0.0001	<0.0001
Arg	167.33^b^	216.16^a^	232.98^a^	78.64^cd^	85.65^c^	84.85^c^	53.45^d^	24.00^e^	58.41^cd^	1.81	<0.0001	0.0040	0.0012
Asp+Asn	27.63^b^	24.65^b^	24.66^b^	31.65^b^	32.95^b^	27.19^b^	393.30^a^	68.01^b^	45.37^b^	2.80	<0.0001	<0.0001	<0.0001
Cit	41.62^d^	47.06^d^	43.56^d^	99.80^a^^b^	104.37^a^	95.76^a^	57.40^c^	38.48^d^	82.74^b^	1.05	<0.0001	0.0260	<0.0001
Cys	64.03^b^	69.17^a^^b^	64.63^a^^b^	45.90^c^	50.19^c^	49.54^c^	72.29^a^^b^	44.72^c^	78.34^a^	1.22	<0.0001	0.2169	0.0007
Cysthi	12.12^bc^	13.03^bc^	13.47^bc^	6.42^c^	10.17^c^	9.73^c^	40.60^a^	7.92^c^	19.60^b^	0.93	<0.0001	0.0094	<0.0001
EOHNH2	52.15^c^	52.64^c^	51.54^c^	48.41^c^	51.18^c^	46.39^c^	171.14^a^	100.57^b^	104.69^b^	1.33	<0.0001	<0.0001	<0.0001
Glu+Gln	521.10^a^	562.39^a^	553.78^a^	373.80^b^	304.86^b^	326.73^b^	326.64^b^	118.22^c^	145.87^c^	3.10	<0.0001	0.0034	0.0013
Gly	314.25^cd^	290.69^d^	327.64^cd^	340.26^cd^	352.60^cd^	344.75^cd^	1503.50^a^	623.93^b^	524.42^bc^	4.81	<0.0001	<0.0001	<0.0001
His	8.69^d^	10.72^d^	8.47^d^	17.62^b^	16.50^b^	18.06^b^	14.14^c^	13.22^c^	23.87^a^	0.52	<0.0001	0.0001	<0.0001
Hylys	16.87^bc^	21.15^b^	18.85^bc^	18.64^bc^	16.00^bc^	10.20^c^	21.16^b^	17.83^bc^	37.04^a^	0.97	0.0002	0.6125	0.0002
Hypro	18.84^e^	19.19^e^	19.35^e^	59.22^cd^	64.70^bc^	72.33^a^^b^	53.13^d^	80.66^a^	78.55^a^	1.12	<0.0001	0.0005	0.0042
Ile	67.37^c^	100.30^a^	90.33^b^	16.36^d^	22.09^d^	23.26^d^	17.40^d^	15.78^d^	22.35^d^	0.91	<0.0001	<0.0001	<0.0001
Leu	58.99^b^	76.91^b^	90.98^b^	47.32^b^	56.37^b^	49.92^b^	278.85^a^	81.26^b^	75.05^b^	2.66	<0.0001	0.0223	<0.0001
Lys	332.53^b^	382.01^a^	363.28^a^	157.12^c^	142.66^cd^	142.86^cd^	134.40^cd^	122.11^d^	152.95^cd^	1.81	<0.0001	0.3098	0.0038
Met	50.86^bcd^	52.52^bc^	54.52^bc^	33.05^e^	36.92^de^	45.47^cde^	74.13^a^	40.21^cde^	60.68^a^^b^	1.26	<0.0001	0.0710	0.0028
Orn	94.67^b^	113.33^a^	118.05^a^	59.89^c^	63.21^c^	48.57^d^	36.35^e^	22.89^f^	54.47^cd^	1.08	<0.0001	0.0063	<0.0001
PEA	16.06^c^	18.18^c^	21.51^c^	17.80^c^	19.94^c^	20.55^c^	247.42^a^	59.56^b^	51.83^b^	1.54	<0.0001	<0.0001	<0.0001
Phe	17.35^cd^	23.08^bc^	26.30^a^^b^	9.90^e^	12.84^de^	12.99^de^	24.35^b^	21.71^bc^	31.65^a^	0.80	<0.0001	0.0006	0.0861
Pro	188.32^a^	157.21^b^	174.86^a^	130.39^c^	137.57^c^	140.21^bc^	93.94^de^	80.24^e^	110.07^d^	1.43	<0.0001	0.0087	0.0095
P-Ser	6.74^c^	6.48^c^	6.95^c^	8.70^c^	9.04^c^	8.29^c^	501.60^a^	70.53^b^	42.58^bc^	2.48	<0.0001	<0.0001	<0.0001
Ser	275.19^bc^	287.33^bc^	343.23^b^	150.42^c^	156.81^c^	146.07^c^	822.22^a^	264.45^bc^	287.21^bc^	4.03	<0.0001	0.0014	<0.0001
Tau	84.69^bc^	93.22^a^^b^	105.82^a^	60.85^d^	61.38^d^	72.38^cd^	59.60^de^	46.83^e^	83.78^bc^	1.23	<0.0001	<0.0001	0.0425
Thr	90.86^c^	100.90^bc^	110.62^bc^	111.47^bc^	127.49^b^	113.51^bc^	177.43^a^	121.43^bc^	201.01^a^	1.86	<0.0001	0.0796	0.0009
Tyr	23.63^bc^	29.72^bc^	39.03^b^	17.16^c^	22.90^c^	19.86^c^	63.59^a^	27.77^bc^	21.31^c^	1.30	0.0003	0.3672	<0.0001
Val	101.02^cd^	119.94^bc^	154.44^a^	84.00^de^	87.83^de^	84.95^de^	128.11^b^	70.93^e^	125.29^b^	1.59	<0.0001	0.0004	<0.0001
α-AAA	6.96^bc^	5.93^c^	3.67^c^	10.35^b^	9.98^b^	6.88^bc^	14.75^a^	17.19^a^	14.73^a^	0.62	<0.0001	0.0119	0.5148
β-AiBA	1.92^d^	2.61^d^	2.52^d^	5.09^d^	0.59^d^	4.91^d^	57.72^a^	19.05^c^	33.06^b^	1.25	<0.0001	0.0158	0.0022
β-Ala	5.99^d^	4.56^d^	4.09^d^	30.35^cd^	23.23^cd^	38.34^bcd^	402.30^a^	89.76^b^	70.03^bc^	2.41	<0.0001	<0.0001	<0.0001
γ-ABA	1.46^c^	1.28^c^	1.33^c^	0.59^c^	0.65^c^	0.96^c^	131.78^a^	32.68^b^	41.18^b^	1.68	<0.0001	0.0004	<0.0001

1 *LBW, the lowest body weight*;

2 *MBW, the middle body weight*;

3 *HBW, the highest body weight*;

a−f *Values within a row without a common superscript letter differ (P < 0.05). Probability values between 0.05 and 0.10 were considered to be trends*.

At day 45 of gestation, HBW fetuses presented higher (*P* < 0.05) amniotic fluid Val concentration compared with the LBW and MBW fetuses. MBW and HBW fetuses presented higher (*P* < 0.05) amniotic fluid concentrations of Lys, Arg, Orn, Phe, and Tau compared with the LBW fetuses; and LBW and HBW fetuses presented higher (*P* < 0.05) amniotic fluid concentrations of Ala and Pro compared with the MBW fetuses. At day 75 of gestation, HBW fetuses presented lower (*P* < 0.05) amniotic fluid Orn concentration compared with the LBW and MBW fetuses, while HBW fetuses presented higher (*P* < 0.05) amniotic fluid Hypro concentration compared with the LBW fetuses. At day 110 of gestation, MBW fetuses presented the highest (*P* < 0.05) amniotic fluid concentrations of Cit, Cysthi, Orn, and β-AiBA, as well as Ans in LBW fetuses; while HBW fetuses presented higher (*P* < 0.05) amniotic fluid concentrations of His, Phe, Ala and Tau compared with the LBW and MBW fetuses. MBW and HBW fetuses presented lower (*P* < 0.05) amniotic fluid concentrations of Leu, Asp, EOHNH2, Gly, Glu, P-Ser, Ser, β-Ala, and γ-ABA compared with the LBW fetuses; while LBW and HBW fetuses presented higher (*P* < 0.05) amniotic fluid concentrations of Met, Thr, Val, and Arg. Finally, HBW fetuses presented higher (*P* < 0.05) amniotic fluid Pro concentration compared with the MBW fetuses.

There were interaction effects (*P* < 0.05) between gestation period and BW on the amniotic fluid concentrations of His, Ile, Leu, Lys, Met, Thr, Val, Ala, Asp, Ans, Cit, Cysthi, Cys, EOHNH2, Gly, Glu, Hylys, Hypro, Orn, Pro, P-Ser, PEA, Ser, Tyr, β-Ala, β-AiBA, and γ-ABA.

### Allantoic Fluid Free AA Profiles in Huanjiang Mini-Pigs During Different Gestation Periods

As showed in Table 7, from day 45 to day 110 of gestation, allantoic fluid concentrations of γ-ABA, Met, and β-Ala were increased (*P* < 0.05) in MBW and HBW fetuses. In addition, the concentrations of Leu and Asp in LBW and MBW fetuses, Hypro in LBW and HBW fetuses, and Val and Orn in HBW fetuses were increased, respectively. The allantoic fluid concentration of Arg was decreased (*P* < 0.05) in the three different BW fetuses, as well as Lys and Orn in the LBW and MBW fetuses and Glu in the MBW fetuses. When compared with day 45 and day 75 of gestation, allantoic fluid concentrations of Ans and Ser were increased (*P* < 0.05) in the three different BW fetuses at day 110 of gestation. Moreover, the concentrations of Phe, Cysthi, EOHNH2, Tyr, 3-Mehis, and β-AiBA in the MBW and HBW fetuses, the concentration of Pro in the LBW and MBW fetuses, and the concentrations of Leu, Asp, and Car in the HBW fetuses, were increased (*P* < 0.05) at 110 days of gestation compared to day 45 and day 75 of gestation. The concentrations of Lys in the HBW fetuses, and Cit corresponding to LBW fetuses were decreased (*P* < 0.05) at day 110 of gestation compared with day 45 and day 75 of gestation. At day 75 of gestation, allantoic fluid concentrations of Cit and Tau in the HBW fetuses and the concentration of β-Ala in the LBW fetuses were increased (*P* < 0.05), whereas allantoic fluid Cys concentration in the HBW fetuses and the concentration of Thr in the LBW fetuses were decreased (*P* < 0.05) compared to the day 45 and day 110 of gestation. Allantoic fluid concentrations of Ala and Gly in the LBW and HBW fetuses were decreased (*P* < 0.05) at day 45 of gestation, as well as the concentration of Pro in the HBW fetuses, and Val and 3-Mehis in the LBW fetuses; whereas the concentrations of Glu in the LBW and HBW fetuses and the concentration of His in the LBW fetuses were increased (*P* < 0.05) when compared with day 75 and day 110 of gestation. Allantoic fluid concentration of Met in the LBW fetuses was increased (*P* < 0.05) at day 75 of gestation, whereas the concentration of Hylys in the HBW fetuses was decreased (*P* < 0.05) at day 110 of gestation, when compared with day 45 of gestation. In comparison with day 75 of gestation, allantoic fluid concentration of His in the MBW fetuses was decreased (*P* < 0.05) at day 110 of gestation, as well as the concentration of Hylys in the LBW fetuses.

**Table 7:** Free AA profiles in allantoic fluid of fetal pigs with LBW, MBW, and HBW at different gestation periods (nmol/ml).

**Items**	**Day 45 of gestation**			**Day 75 of gestation**			**Day 110 of gestation**			**SEM**	***P*****-values**		
	**LBW^**1**^**	**MBW^**2**^**	**HBW^**3**^**	**LBW**	**MBW**	**HBW**	**LBW**	**MBW**	**HBW**		**Gestation period**	**Body weight**	GP*BW
3-Mehis	4.55^d^	3.34^d^	4.19^d^	18.05^bc^	12.19^cd^	11.69^cd^	22.94^b^	39.73^a^	25.03^b^	0.95	<0.0001	0.1053	0.0057
Ala	168.15^b^	175.20^b^	263.48^b^	496.34^a^	215.46^b^	525.54^a^	437.97^a^	470.99^a^	541.04^a^	3.50	<0.0001	<0.0001	0.0006
Ans	215.39^de^	137.96^e^	149.06^e^	327.18^de^	377.02^d^	305.93^de^	1029.30^c^	1640.98^a^	1277.49^b^	4.40	<0.0001	0.0002	0.0004
Arg	1145.05^a^^b^	999.51^b^	1317.74^a^	429.22^c^	425.58^c^	401.06^c^	79.63^d^	114.32^d^	117.46^d^	5.14	<0.0001	0.4345	0.2762
Asp+Asn	155.40^de^	145.37^de^	95.09^e^	273.57^cd^	344.01^c^	231.47^cde^	595.34^b^	848.66^a^	839.94^a^	4.02	<0.0001	0.0723	0.0346
Car	22.97^abc^	16.24^cd^	15.18^cd^	18.40^bcd^	21.43^abcd^	13.68^d^	17.63^cd^	26.15^a^^b^	29.06^a^	0.90	0.0032	0.0147	0.0414
Cit	109.11^abc^	101.06^bcd^	119.95^a^^b^	120.81^a^^b^	110.90^abc^	133.06^a^	74.09^d^	88.30^cd^	82.22^cd^	1.74	<0.0001	0.8199	0.2342
Cys	109.37^bc^	110.59^bc^	136.52^a^^b^	88.59^cd^	105.68^bc^	68.79^d^	95.77^cd^	156.23^a^	136.99^a^^b^	1.92	<0.0001	0.0393	0.0131
Cysthi	18.72^b^	18.26^b^	19.76^b^	22.47^b^	29.32^b^	20.71^b^	24.95^b^	82.60^a^	82.41^a^	1.20	<0.0001	<0.0001	<0.0001
EOHNH2	141.43^b^	106.70^b^	142.55^b^	114.28^b^	106.49^b^	103.53^b^	90.27^b^	198.85^a^	207.24^a^	2.24	<0.0001	0.1072	0.0003
Glu+Gln	1039.51^a^	841.32^a^	918.45^a^	432.96^bc^	283.92^c^	440.15^bc^	414.77^bc^	506.41^b^	485.25^bc^	4.56	<0.0001	0.1767	0.2229
Gly	310.93^c^	384.65^c^	389.47^c^	2086.50^a^^b^	675.18^c^	1950.33^a^^b^	1656.23^b^	2411.81^a^	2050.29^a^^b^	7.57	<0.0001	0.0229	<0.0001
His	68.62^a^	43.61^bc^	31.96^b^	42.30^bc^	50.76^b^	41.80^bc^	36.61^bc^	30.28^c^	35.29^bc^	1.29	0.0069	0.0200	0.0035
Hylys	242.52^a^^b^	259.34^a^	218.11^abc^	239.37^a^^b^	272.45^a^	202.82^abc^	122.12^c^	254.23^a^	135.54^bc^	1.96	0.0537	0.0699	0.3265
Hypro	20.38^d^	16.97^d^	19.06^d^	56.99^c^	17.99^d^	57.59^c^	73.92^b^	49.95^c^	93.12^a^	1.29	<0.0001	<0.0001	0.0001
Leu	45.52^d^	55.23^d^	42.62^d^	135.29^c^	135.29^c^	90.36^cd^	388.40^b^	566.05^a^	566.39^a^	2.69	<0.0001	0.0175	0.0002
Lys	1093.14^a^^b^	1136.79^a^	950.69^b^	727.52^c^	576.47^d^	1024.31^a^^b^	295.71^e^	402.78^e^	288.70^e^	4.04	<0.0001	0.0894	<0.0001
Met	20.70^d^	23.99^d^	19.50^d^	50.19^c^	45.55^c^	49.40^c^	36.68^cd^	157.38^a^	117.20^b^	1.40	<0.0001	<0.0001	<0.0001
Orn	1728.79^a^	1438.24^b^	1580.90^a^^b^	725.66^c^	611.13^c^	604.45^c^	41.00^d^	187.58^d^	62.64^d^	4.93	<0.0001	0.1230	0.2306
Phe	13.92^cd^	12.15^cd^	9.66^d^	19.18^cd^	20.11^cd^	17.21^cd^	28.32^c^	92.42^b^	158.95^a^	1.30	<0.0001	<0.0001	<0.0001
Pro	79.24^cd^	78.65^cd^	44.51^e^	109.77^bc^	55.15^de^	102.16^bc^	154.70^a^	143.49^a^	130.71^a^^b^	1.84	<0.0001	0.0188	0.0036
P-Ser	71.89^c^	54.01^c^	68.29^c^	176.32^bc^	206.47^b^	136.41^bc^	730.23^a^	809.14^a^	825.88^a^	3.50	<0.0001	0.4839	0.4644
Ser	379.49^cde^	300.83^de^	323.32^de^	549.13^c^	221.81^e^	458.05^cd^	1335.45^a^^b^	1435.44^a^	1243.92^b^	4.35	<0.0001	0.0638	0.0038
Tau	349.08^b^	343.17^b^	329.67^b^	342.35^b^	302.09^b^	556.4^a^	248.63^b^	332.91^b^	345.04^b^	4.06	0.0998	0.0363	0.0638
Thr	320.20^bcd^	235.03^de^	290.52^cd^	187.68^e^	158.76^e^	197.72^e^	348.01^bc^	476.13^a^	389.49^b^	3.01	<0.0001	0.9574	0.0113
Tyr	35.89^cd^	33.74^cd^	24.83^d^	37.55^cd^	48.42^cd^	24.50^d^	55.79^c^	123.71^b^	157.34^a^	1.63	<0.0001	0.0027	<0.0001
Val	62.41^de^	67.38^de^	55.16^e^	134.66^b^	94.38^cde^	102.13^bcd^	131.45^bc^	287.93^a^	269.84^a^	2.01	<0.0001	0.0083	<0.0001
α-AAA	17.27^c^	17.69^c^	24.98^bc^	29.92^bc^	30.06^bc^	26.82^bc^	47.67^b^	129.84^a^	33.59^bc^	1.66	<0.0001	0.0002	<0.0001
β-AiBA	27.75^c^	25.36^c^	33.00^c^	30.26^c^	34.55^c^	35.26^c^	17.45^c^	118.94^a^	95.51^b^	1.48	<0.0001	<0.0001	<0.0001
β-Ala	41.39^c^	31.80^c^	33.77^c^	184.09^b^	189.68^b^	182.14^b^	38.30^c^	929.10^a^	955.60^a^	2.80	<0.0001	<0.0001	<0.0001
γ-ABA	10.13^e^	8.51^e^	6.56^e^	32.44^d^	57.94^c^	32.61^d^	39.11^d^	309.30^b^	440.02^a^	1.26	<0.0001	<0.0001	<0.0001

1 *LBW, the lowest body weight*;

2 *MBW, the middle body weight*;

3 *HBW, the highest body weight*;

a−e *Values within a row without a common superscript letter differ (P < 0.05). Probability values between 0.05 and 0.10 were considered to be trends*.

At day 45 of gestation, HBW fetuses presented lower (*P* < 0.05) allantoic fluid concentrations of Lys and Pro compared with the LBW and MBW fetuses; MBW and HBW fetuses presented lower (*P* < 0.05) allantoic fluid His concentration compared with the LBW fetuses; and HBW fetuses presented higher (*P* < 0.05) allantoic fluid Arg concentration compared with the MBW fetuses. At day 75 of gestation, HBW fetuses presented higher (*P* < 0.05) allantoic fluid Tau concentration compared with the LBW and MBW fetuses; while LBW and HBW fetuses presented higher (*P* < 0.05) allantoic fluid concentrations of Ala, Gly, Hypro, Pro, and Ser. Lower (*P* < 0.05) allantoic fluid γ-ABA concentration was measured, when compared with the values recorded in the MBW group of fetuses. In addition, HBW fetuses presented lower (*P* < 0.05) allantoic fluid Cys concentration compared with the MBW fetuses. At day 110 of gestation, HBW fetuses presented the highest (*P* < 0.05) allantoic fluid concentrations of Phe, Tyr, and γ-ABA; while HBW fetuses presented lower (*P* < 0.05) allantoic fluid Val concentration compared with the LBW and MBW fetuses. MBW and HBW fetuses presented higher (*P* < 0.05) allantoic fluid concentrations of Leu, Asp, Car, Cysthi, Cys, and β-Ala compared with the LBW fetuses; while LBW and HBW fetuses presented higher (*P* < 0.05) allantoic fluid concentration of Hypro. Lower (*P* < 0.05) allantoic fluid concentrations of Thr, Hylys, and α-AAA were recorded, when compared with the MBW fetuses. In addition, HBW fetuses presented lower (*P* < 0.05) allantoic fluid Ser concentration compared with the MBW fetuses.

There were interaction effects (*P* < 0.05) between gestation period and BW on the allantoic fluid concentrations of His, Leu, Lys, Met, Phe, Thr, Val, Ala, Asp, Ans, Car, Cysthi, Cys, EOHNH2, Gly, Hypro, Pro, Ser, Tyr, 3-Mehis, α-AAA, β-AiBA, and γ-ABA.

## Discussion

The physiological status is reflected by the metabolic profile (21). Amniotic and allantoic fluids are crucial for physiological exchanges between fetal and maternal tissues, of which biochemical parameters and free AAs could mirror the nutritional transportation from the mother to the fetus in the process of fetal growth and development (22). Amniotic fluid is the inner environment of fetal life and contains large amounts of protein and metabolites produced by the amnion epithelial cells, fetal tissues, fetal excretions, and placental tissues (23, 24). The allantoic fluid is also considered as a factor affecting the fetal growth and development due to the nutrients it contains (15). The present study was aimed to analyze the biochemical parameters and AAs concentration of maternal plasma, amniotic fluid, and allantoic fluid to identify the nutrients that may affect fetal growth and development. Our findings show that the metabolite profiles in biological fluids recovered from fetuses with different BW fetuses are markedly distinct and suggest that Arg, Gln, Glu, Pro, Tau, and His are AAs that may play major functions for fetal growth and development.

The ALT and AST play a central role in the transamination and reflect the status of protein synthesis and catabolism. The increase in plasma enzymatic activity appears related to improved AA metabolism (25). The ALB is one of the major endogenous proteins, which represents an indicator of the body's absorption and metabolism of AAs. In the present study, increases in the ALT activity and ALB concentration suggest that the mothers improve their AA metabolism as gestation progressed presumably in order to satisfy the nutritional needs of fetuses. The plasma concentrations of TC, TG, HDL-C, and LDL-C are the pivotal indexes to measure the intensity of lipid metabolism of the body. The CHE activity is related to lipid metabolism, and the increased CHE activity suggests an increased lipid metabolism (26). The increased TG concentration indicates that fat deposition decreases and HDL-C and LDL-C concentrations are known to be relevant to the transportation of lipids (27). In the present study, the plasma TG concentration at day 110 of gestation was the highest, whereas plasma concentration of HDL-C and activity of CHE was the lowest, suggesting that lipid metabolism and deposition of pregnant Huanjiang mini-pigs declined as gestation advanced due to the decreasing fat deposition of sow during the third trimester of pregnancy (28). This is related to the maximal fetal growth occurring during the third trimester of pregnancy, in a context of increased nutrient needs for the fetuses (29). The AMM and UN concentrations accurately reflect the status of protein metabolism and AA balance in the animal body. This study showed that AMM concentration decreased, demonstrating that sows likely improve protein utilization as the pregnancy progressed. The study by Cappai and collaborators also showed that protein metabolism is higher in goats during late pregnancy due to the need for fetal-placental development (30). The ALP in the blood and bone is involved in the formation of hydroxyapatite, which is important for regulating ordered mineral deposition during bone formation (31). In accordance with van Riet's reports, the present study also showed that ALP activity was the highest at day 75 of gestation (32). These are mainly relevant to the growth and development of the fetal skeletal system. As found in the present study, the TBA concentration increased as gestation advanced, and this may be due to the increased bile acids transport in the fetus-to-mother direction in the third trimester (33, 34).

Study has demonstrated that the protein and metabolite composition of amniotic fluid varies throughout gestation, such as γ-glutamyl transferase, urea, and creatinine (35). The increased levels of enzymes in the later period of gestation correlate with the formation of fetal kidneys, lungs, and gastrointestinal tract (35). In the present study, the amniotic fluid activities of AST and CHE and concentrations of TP and UN were the highest regardless of the BW at day 110 of gestation, as well as allantoic fluid CHE activity and TP concentration, suggesting that an increase in fetus function enhances the metabolism of lipids and amino acids. Fetal urine contributes to allantoic fluid (36). This could explain the allantoic fluid UN concentration increased regardless of the BW at day 75 and day 110 of gestation. The AST and ALT act as a catalyst in connecting the metabolism of amino acids and carbohydrates, and changes in metabolic activity may reflect the metabolic events observed. As observed in the present study, the decreased activity of AST in allantoic fluid recovered from LBW and HBW fetuses as gestation progressed, is in accordance with a previous study in ewes (37). The allantoic fluid activity of AST corresponding to MBW fetuses was the lowest at day 75 of gestation, whereas those corresponding to the LBW fetuses were the highest. This is possibly associated with the immature liver of LBW fetuses, which is not functioning adequately. The AMM is produced by deamination of amino acids, which is transformed to urea by liver, and then excreted by kidney. The allantoic fluid concentration of AMM regardless of the BW was the lowest at day 110 of gestation, which may be due to fetal liver and kidney is already mature (38), and then the AMM could be transformed to urea to be excreted. The amniotic fluid AMM concentration corresponding to LBW and MBW fetuses were the highest at day 110 of gestation, which may be due to the quantities swallowed by the LBW and MBW fetuses, those are lower than the quantity swallowed by the HBW fetuses. The allantoic fluid GLU concentration corresponding to three different BW fetuses increased as gestation advanced in the present study. This result is in agreement with the report by Zanella et al. (39) in mares, but different from the reports by Khatun et al. (40) in sheep and Tabatabaei et al. (38) in cattle, which indicated that allantoic fluid glucose concentration presented a decreasing trend with the advanced pregnancy. From early gestation, the fetuses can produce bile acids and bilirubin (33). The present study indicated that the allantoic fluid concentrations of TBA and BIL increased regardless of the BW as gestation advanced, due to the strengthened metabolism of TBA and BIL in the gradually mature liver.

The amniotic fluid TBA concentration corresponding to LBW fetuses was the lowest at day 45 of gestation due to the fact that their liver likely secretes less TBA, whereas HBW fetuses had the highest TBA concentration at day 110 of gestation due to their fully developed liver function. The allantoic fluid UN concentration in LBW fetuses was the lowest at day 75 and day 110 of gestation, demonstrating that the protein metabolism in LBW fetuses was weakened. In the present study, the allantoic fluid AMM concentration in LBW fetuses was the lowest at day 45 and day 75 of gestation due to the fact that LBW fetuses display weak deamination of amino acids, whereas the HBW fetuses had the lowest AMM concentration at day 110 of gestation, suggesting that the liver of HBW fetuses could efficiently transform AMM to urea. The allantoic fluid concentrations of TBA and BIL in HBW fetuses were the lowest at day 110 of gestation, demonstrating that the HBW fetuses likely excrete TBA and BIL more efficiently by placenta to maintain a lower levels in the fetal compartment (33).

Maternal metabolism undergoes a series of metabolic adaptions during pregnancy to sustain the growth of the fetus and placenta (41). During pregnancy, AAs represent one of the major nutrients and are important precursors for fetal development and growth, for the biosynthesis of compounds including protein, nucleotides, neurotransmitters, polyamines, and nitric oxide (42, 43). In the present study, the plasma concentrations of Asp, Cys, Cit, Glu, Hypro, Leu, Pro, PEA, Phe, Sar, and Ser were the highest at day 45 of gestation, as well as those of Lys, Val, EOHNH2, Hylys, Tau, 1-Mehis, and β-Ala at day 75 of gestation, and those of Ile, Car, Cysthi, His, Orn, 3-Mehis, Tyr, α-AAA, α-ABA, and β-AiBA at day 110 of gestation. These findings reinforce the view that the sows need different AAs to satisfy the growth and development of the placenta and fetus during gestation, due to the growth of the placenta, fetus, and fetal tissues occurring at different rates during different gestation periods (44).

McPherson et al. (44) indicated that the composition of individual fetal tissues undergoes dynamic changes during gestation, suggesting that maternal and fetal nutrient requirement varies with the gestation period. Besides glucose, fatty acids, and lactate, the AAs are crucial for fetal growth and development, which are not only an important energy source for growing fetuses but also the precursor of protein synthesis (45, 46). Fetal growth depends upon the utilization of free AAs in the synthesis of structural and functional proteins. In the present study, the amniotic fluid concentrations of Hypro, EOHNH2, PEA, α-AAA, β-AiBA, β-Ala, and γ-ABA in three different BW fetuses increased as gestation progressed, as well as the allantoic fluid concentrations of Leu, Val, Asp, Hypro, P-ser, γ-ABA, Ala, Ans, Gly, Pro, Ser, and 3-Mehis. This may result from the dwindling demand of these AAs for fetal development at the third trimester pregnancy. The amniotic fluid concentrations of Ile, Lys, Ala, Arg, Glu, Orn, Pro, and Tau in the three BW fetuses decreased as gestation progressed, as well as allantoic fluid concentrations of Lys, Arg, and Glu. This is likely associated with increased fetal demands of elementary building blocks, which are necessary for protein synthesis to support the very rapid growth of fetuses (47). Arg, Orn, Pro, and Glu belong to functional AAs and Arg can act as precursors of Orn, Pro, and Gln, all of which are crucial for fetal development (48). Several studies have indicated that Arg, Gln, Glu, and Pro modulate gene expression and enhance the growth of the small intestine (49–51). Indeed the intestinal growth accelerates during late gestation (44). In addition, Tau is considered an essential AAs for the fetuses and neonates, and *de novo* fetal synthesis is known to be inadequate at these ages (52). In the present study, there were different changes among three different BW fetuses in the amniotic fluid concentrations of His, Leu, Thr, Asp, Cysthi, Gly, Hylys, P-Ser, Ser, Tyr, Met, Car, and Val, as well as allantoic fluid concentrations of Met, Orn, β-Ala, Phe, Thr, Car, Cysthi, Cys, EOHNH2, Hypro, Tyr, α-AAA, β-AiBA, Cit, His, Hylys, Tau, and Met. These results may be relevant to the distinct digestion and metabolism of nutrients among three different BW fetuses. Further works are required to progress on that aspect.

The dynamic changes of AAs in fetal fluids according to different BW fetuses suggest strongly that the different BW fetuses have distinct nutrient requirements due to their different developmental trajectories. A previous study indicated that the marked changes in concentrations of AAs in ovine amniotic and allantoic fluids were associated with conceptus development (12). Amniotic fluid is a vital source of nutrients for the gut and other fetal tissues, which provides amino acids and other substances for supporting the proliferation and differentiation of intestinal epithelial cells (15). At day 45 of gestation, the amniotic fluid concentrations of Lys, Arg, Orn, Phe, Tau, and Val in the HBW fetuses were higher than the LBW fetuses; and the amniotic fluid concentrations of Ala and Pro in the MBW fetuses were the lowest. The importance of Lys in fetal growth may be relevant to its crucial regulatory role in nitric oxide synthesis, protein methylation, acetylation, ubiquitination, and O-linked glycosylation (53). Arg is nutritionally essential for fetal-placental growth and development *via* its role in nitric oxide signaling and polyamine synthesis (54). In brief, the higher amniotic fluid concentrations of Lys, Arg, Orn, Phe, Tau, and Val in the HBW fetuses are likely related to the more rapid development in this group of animals than in the LBW fetuses group during early gestation. With the development of intestinal AA transport systems during gestation, the swallowing of amniotic fluid provides a source of AAs for fetal utilization (55). At day 110 of gestation, the amniotic fluid concentrations of His, Phe, Ala, Tau, and Hypro in the HBW fetuses were higher than the LBW fetuses; the amniotic fluid concentrations of Leu, Asp, EOHNH2, Gly, Glu, P-ser, Ser, β-Ala, PEA, and γ-ABA in the LBW fetuses were highest. These data demonstrated that the HBW fetuses develop faster, whereas the LBW fetuses need more AAs to support their growth and development due to the slower development during the late gestation. For example, Glu is an abundant amino acid in amniotic fluid and is an important nutrient that play important role as fuel for enterocytes, and as precursor of metabolites with regulatory roles (56), and stimulates intestinal growth and development (12). In the present study, amniotic fluid Glu concentration in the LBW fetuses was higher than the HBW and MBW fetuses, indicating that the LBW fetuses need more Glu on account of those delayed growth and development.

Previous study has shown that total recoverable amounts of Arg, His, Orn, and Lys increased by 8-, 22-, 5-, and 28-fold, respectively (57), indicating that these AAs play a vital role in the growth and development of the fetuses. At day 45 of gestation, the allantoic fluid concentrations of His, Lys, and Pro in the HBW fetuses were lower than the LBW fetuses, mainly due to the fact that accumulation of allantoic fluid during early gestation is probably linked to the transmembrane transport and secretory activity of the extra-embryonic membranes (58) and to the fact that the HBW fetuses likely absorb more His, Lys, and Pro during the early gestation. In the present study, the allantoic fluid Arg concentration in the HBW fetuses at day 45 of gestation was higher than the MBW fetuses. Wu et al. also reported that Arg is abundant in porcine allantoic fluid during early gestation (42). These findings suggest that the higher Arg concentration is beneficial for the HBW fetal development during early gestation. Allantoic fluid nutrients could be absorbed by the allantois into the fetal-placental circulation and utilized by fetal tissues, in accordance with the critical role of allantois in the fetal nutrition (15). At day 75 of gestation, the allantoic fluid concentrations of Lys, Val, Ala, Gly, Hypro, Pro, and Ser in the MBW fetuses were lower than the HBW and LBW fetuses, suggesting that these AAs were absorbed more efficiently by the MBW fetuses to maintain their growth at the mid-trimester of pregnancy. For example, Ala, Gly, Pro, and Ser have a positive effect on the fetal growth (12, 50). At day 110 of gestation, the allantoic fluid concentrations of Leu, Asp, Car, Cysthi, Cys, Phe, Tyr, β-AiBA, and β-Ala in the HBW and MBW fetuses were higher than the LBW fetuses; and the allantoic fluid concentrations of Met, Thr, Hylys, Ans, α-AAA, and β-AiBA in the MBW fetuses were higher than the HBW and LBW fetuses. These results may explain why the LBW fetuses develop slower, and in turn the undeveloped organ affects the metabolism of fetuses and nutrient composition of the allantoic fluid.

## Conclusions

As the gestation progressed, the pregnant Huanjiang mini-pigs present signs of improved utilization of protein and declined lipid metabolism and deposition, the fetuses present signs of enhanced metabolism of lipids and amino acids, and the HBW fetuses display signs of stronger ability for protein synthesis. The Arg, Gln, Glu, Pro, Tau, and His, although considered as non-essential amino acids, appears as likely central for fetal growth and development. These findings indicate that the metabolite profiles of maternal plasma and fetal fluids show remarkable changes. It is tempting to propose that such changes may correspond to an adaptation to rapid fetal growth and development as gestation advanced. These may illustrate that the metabolite profiles of different BW fetuses have markedly distinct features. Further studies, notably with nutrients labeled with stable isotopes, are needed to identify the metabolic pathways participating in these changes.

## Data Availability Statement

The original contributions presented in the study are included in the article/supplementary material, further inquiries can be directed to the corresponding author.

## Ethics Statement

The animal study was reviewed and approved by Animal Care and Use Committee of the Institute of Subtropical Agriculture, Chinese Academy of Science. Written informed consent was obtained from the owners for the participation of their animals in this study.

## Author Contributions

QZ and XK performed sampling and nutrient measurements, analyzed data and interpreted the results, and drafted the manuscript. PX conducted animal feeding and sampling. HL participated in the nutrient measurements. XK and YY contributed to experimental concepts and design, provided scientific direction, and together with FB finalized the manuscript. All authors read and approved the final manuscript.

## Conflict of Interest

The authors declare that the research was conducted in the absence of any commercial or financial relationships that could be construed as a potential conflict of interest.
